# The larval chondrocranium and its development in *Smilisca phaeota* with considerations of patterns characteristic for the chondrocranial development of Lalagobatrachia

**DOI:** 10.1038/s41598-024-70724-9

**Published:** 2024-08-26

**Authors:** Janine M. Ziermann-Canabarro, Paul Lukas

**Affiliations:** 1 Howard University College of Medicine, 520 W St NW, Washington, DC 20059 USA; 2 Institute of Zoology and Evolutionary Research, Friedrich-Schiller-University, Jena, Germany

**Keywords:** Pharyngeal arch, Neurocranium, Viscerocranium, Developmental pattern, Developmental sequence, Herpetology, Cartilage development, Evolutionary developmental biology

## Abstract

Several studies describe the development of the chondrocranium of vertebrates. The details in these studies vary a lot, which makes it hard to compare developmental patterns and identify evolutionary trends. Therefore, we aim to close this gap for anurans, which is the largest order of amphibians. We present here a detailed description of the chondrocranium morphology and development of *Smilisca phaeota,* the New Granada cross-banded tree frog. The anatomy was described for the larvae at or older than Gossner stage 31 and before ossification starts. Following this, we describe the development of the chondrocranium from Gossner stages 19–26. Early in Gossner stage 19 no precursors of any cartilages are visible, while later in that stage the mesodermal Anlage of Meckel’s cartilage was observed. In the subsequent stages more and more mesodermal anlagen become identifiable, followed by chondrification, and final differentiation of the cartilage elements. We used serial sections to study all the developmental stages and additionally utilized cleared and stained specimens and CT scan data. The latter were also used for the 3D reconstruction of the chondrocranium. We previously studied several species and compared these developmental patterns with *S. phaeota*, revealing potentially characteristic patterns significant for Lalagobatrachia, a clade that includes over 7000 frog species. These include (1) the suprarostral alae develop before the suprarostral corpus, (2) the infrarostral cartilage chondrifies late, after the chondrification of ceratobranchial 1, and (3) the ceratohyal body is the first element to show chondrocytes and to chondrify. However, with only six species studied so far, our data only provide a basis for future studies and developing hypotheses about the ancestral developmental pattern in anurans.

## Introduction

The vertebrate head development is complex, resulting in a species-specific structure comprising cartilage, muscles, vessels, and nerves, and often also bone. There is a huge interest into the evolution and development of the chondrocranium, i.e., the cartilaginous part of the skull, which is the first part to develop and evolve in vertebrate skulls^1–3^. It is also important as it contains the brain, major sensory systems, feeding apparatus, and the beginning of the respiratory system^4^.

Adaptations to different environments, feeding types, defense needs, etc., resulted in a large diversity of vertebrate skulls^5,6^. Despite this, there are general patterns and structures found in almost all vertebrates^7,8^. For example, the Meckel’s cartilage will form the lower jaw or the template for the later evolving and developing mandible^9^. The skull can be subdivided into the neurocranium, the parts surrounding the brain, and the viscerocranium (also called splanchnocranium or branchiocranium), which forms the jaws and skeletal elements anterior to the neurocranium that are derived from pharyngeal arch (PA) cartilaginous elements^10,11^. The chondrocranium is in many adult species (ostheichthyes, tetrapods) partially or completely replaced by bone.

To identify general patterns and differences during the evolution and development of the vertebrate skull, it would be ideal to study it in representatives of all genera or at least all families. Since this is not possible, selecting a clade of vertebrates with diverse skulls enables researchers to evaluate which parts of the skull are more evolvable, i.e., change faster or are more likely to change, and which parts are more conserved. Therefore, identifying parts less constrained in a diverse group will provide the development of testable hypotheses in other taxa and in larger groups to study the evolutionary development of the vertebrate skull.

One diverse group is the amphibian order Anura (frogs and toads). There are currently 7693 anuran species in over 50 families known^12^. The families can be grouped into suborders archeobatrachians, mesobatrachians, and neobatrachians, with the first considered to be the evolutionary oldest and the last the evolutionary youngest group. Mesobatrachians forming an evolutionary link between the two other groups^13^. There are only a few studies on the chondrocranial development in the diverse anuran larvae, which is a missed opportunity to analyze evolutionary and developmental patterns. Chondrocrania in anuran larvae are very diverse, ranging from miniaturized predators that have formed a suction tube with their jaw elements (e.g., Hymenochirus boettgeri), to giant heads with a wide gaping mouth (e.g., Lepidobatrachus laevis), with all kinds of shapes (slender, wide, short, long, etc.) inbetween. The chondrocranial development of *Ascaphus truei* (archaeobatrachian), was partially described^14^. Lukas and colleagues have described the chondrocranial development in X*enopus laevis* (mesobatrachian)*, Bufo bufo* (neobatrachian)*,* and *Bombina orientalis* and *Discoglossus scovazzi* (both archeobatrachian)^15–18^.

Neobratrachian frogs are the most diverse and species rich group of anurans, but they are underrepresented in the most recent studies. The hylid family includes 1048 known species^12^. They are tree frogs populating the Americas, Australo-Papunea, and temperate Eurasia^19,20^. Hylinae is the largest subfamily containing 2/3rds of the hylid species^12,20^. The monophyly of Middle American and Holarctic Hylinae is mainly due to biogeographic reasons^21^ and is supported by a large genetic study^22^. The genus *Smilisca comprises 9 species*^12^* and* is part of this Middle American Hylinae group. Our species of interest here, *Smilisca phaeota* (New Granada cross-banded tree frog), can be found in humid forests from Nicaragua to northern Colombia^21^.

Since recent studies on chondrocranial development focused on Archaeobatrachia, we aim to add here the neobatrachian species *Smilisca phaeota*. Hypotheses from previous studies will be tested by comparing them to S. phaeota’s development. The description of the chondrocranium and its development in *S. phaeota* is therefore one step in a larger project to achieve a better understanding of the evolutionary development of the chondrocranium in anurans and ultimately in vertebrates.

## Results

Pharyngeal arches (PAs) are bilateral, lateroventral pockets that contain a mesenchymal core of mesodermal and neural crest cells. Each PA mesenchyme develops arch-specific cartilages. These are from anterior to posterior PAs:

- PA1 (mandibular ach, MA): infrarostral, suprarostral, palatoquadrate, and Meckel’s cartilage;
- PA2 (hyoid arch, HA): ceratohyal;
- PA3-6 (branchial arches, BAs 1–4): ceratobranchialia, hypobranchial, and commissurae between the ceratobranchialia.

Cartilages surrounding the brain belong to the neurocranium.

In order to better understand the stages of early chondrogenesis and to introduce the corresponding structures as they are fully developed, we will start with the description of the chondrocranium at stage Go 31, before describing its development in the following section.

### Chondrocranium of larval *Smilisca* phaeota at stage Go 31 (Fig. 1)

**Fig. 1:**
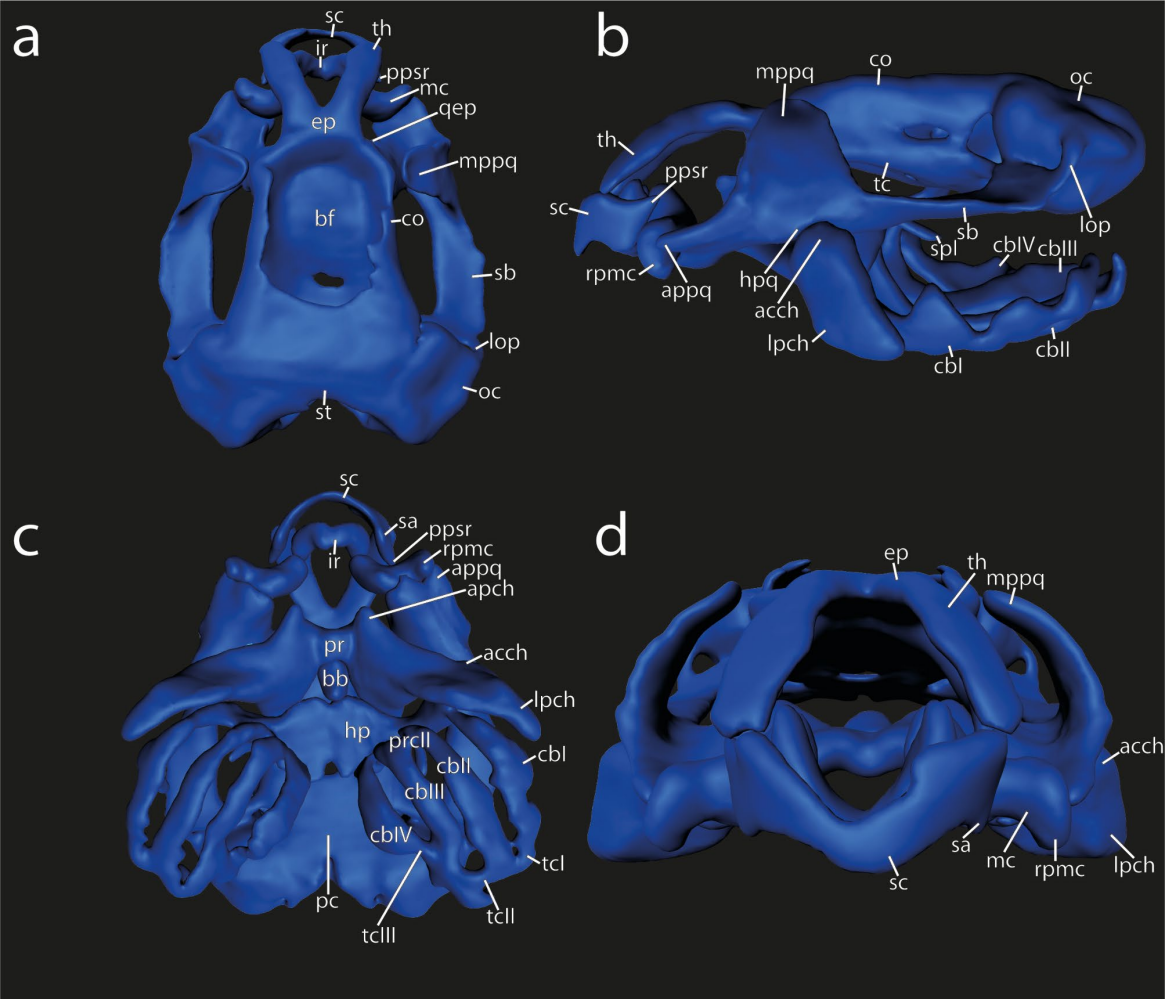
3D reconstructions of the chondrocraniumof *Smilisca phaeota* larvae at Gosner stage 35 in (**a**) dorsal view (without ceratohyal and branchial basket), (**b**) lateral view, (**c**) ventral view (anterior to the left), and (**d**) frontal view. Abbreviations: acch, articular condyle of ceratohyal; apch, anterior process of ceratohyal; appq, articular process of palatoquadrate; bb, basibranchial; bf, basicranial floor; cbI‐IV, ceratobranchial I–IV; co, orbital cartilage; ep, ethmoid plate; hp, hypobranchial plate; hpq, hyoquadrate process of palatoquadrate; ir, infrarostral cartilage; lop, larval otic process; lpch, lateral process of ceratohyal; mc, Meckel's cartilage; mppq, muscular process of palatoquadrate; oc, otic capsule; pc, parachordal cartilage; ppsc, posterior process of suprarostral cartilage; pr, pars reuniens; prcII, proximal commissure II; qep, quadratoethmoid process; rpmc, retroarticular process of Meckel´s cartilage; sa, suprarostral ala; sb, subocular bar; sc, suprarostral corpus, spI, spicule I; tc, trabecular cartilage; tcI-III, terminal commissure I–III; th, trabecular horn.

**Suprarostral cartilage** formed by a single element; slender U-shaped corpus; suprarostral alae bordering trabecular horns ventrally, ventral margin convex, dorsal margin concave; posterior process present.

I**nfrarostral cartilages** U- shaped in anterior view; medial synchondrosis; articulates with Meckel’s cartilage at the intramandibular joint.

**Meckel’s cartilage** sigmoidal; semicircular ventromedial process is anteromedial located; retroarticular process extends posterolaterally, forming the primary jaw joint with the articular process of the palatoquadrate.

**Palatoquadrate** with broad anterior quadratocranial commissure, narrow ascending process, and dorsoventrally extending larval otic process; quadratoethmoid process present; muscular process broad, dorsal tip bends medially to the lateral brain wall; hyoquadrate process slightly bulged ventrally; subocular bar broad, flat, and oblique.

**Basihyal** absent.

**Ceratohyalia** are dorsoventrally depressed horizontal plates, lateral parts are rotated 90 degrees in the transverse plane; anterior process triangular; lateral process wider than subocular bar; articular condyle semicircular; posterior process triangular; medial pars reuniens; basibranchial confluent with pars reuniens, bears pointed urobranchial process.

**Hypobranchial plate** wider than long, anterior margin with two protrusions, posterior margin bears two pointed tubercles.

**Ceratobranchial** I connected to hypobranchial plate, bears pointed spicule; ceratobranchials II–IV not connected to hypobranchial plate, no spicules; proximal commissures I–III present; terminal commissure I–III present; craniobranchial commissures absent.

Anterior **trabecular horns** same plane as infrarostral cartilage, quarter circle in lateral view; ethmoid plate short; lateral brain wall consists of **orbital cartilage** and **marginal tectal taenia,** optic and oculomotor foramen present; **basicranial floor** fully chondrified; **synotic tectum** connects otic capsules dorsally; **medial tectal taenia** present; **transversal tectal taenia** connecting the marginal and medial tectal taenia, two circular parietal fenestra (pers. obs. in Go 35 specimens).

### Chondrocranial development of *Smilisca* phaeota (Table 1)

**Table 1:**
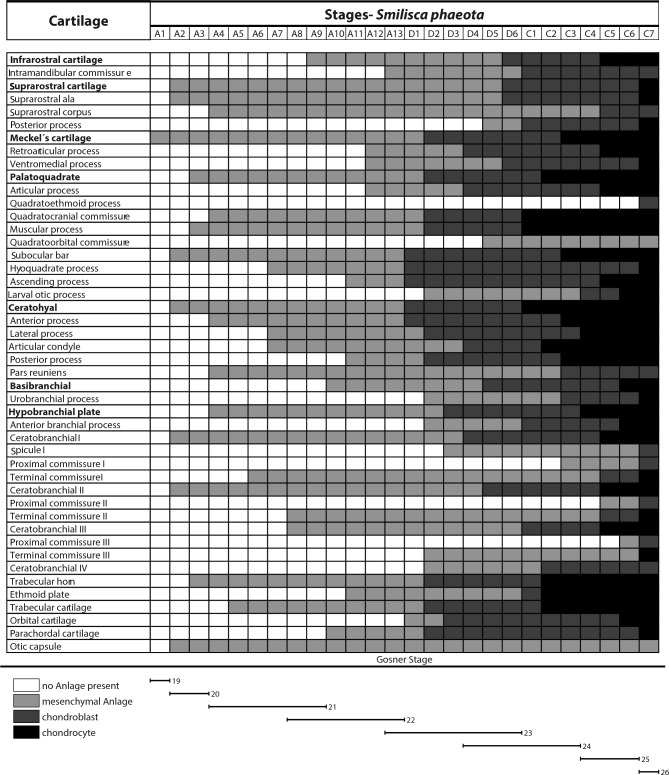
Sequence of development of cartilage in *Smilisca phaeota*. Cranial structures are shown on the left, and developmental stages are shown on the subsequent columns. Light gray represents visible mesenchymal Anlage, dark gray represents chondroblasts, and black represents cartilage. Lines below the table mean the equivalence to Gosner (1960) staging.

#### Infrarostral cartilage

**Fig. 2:**
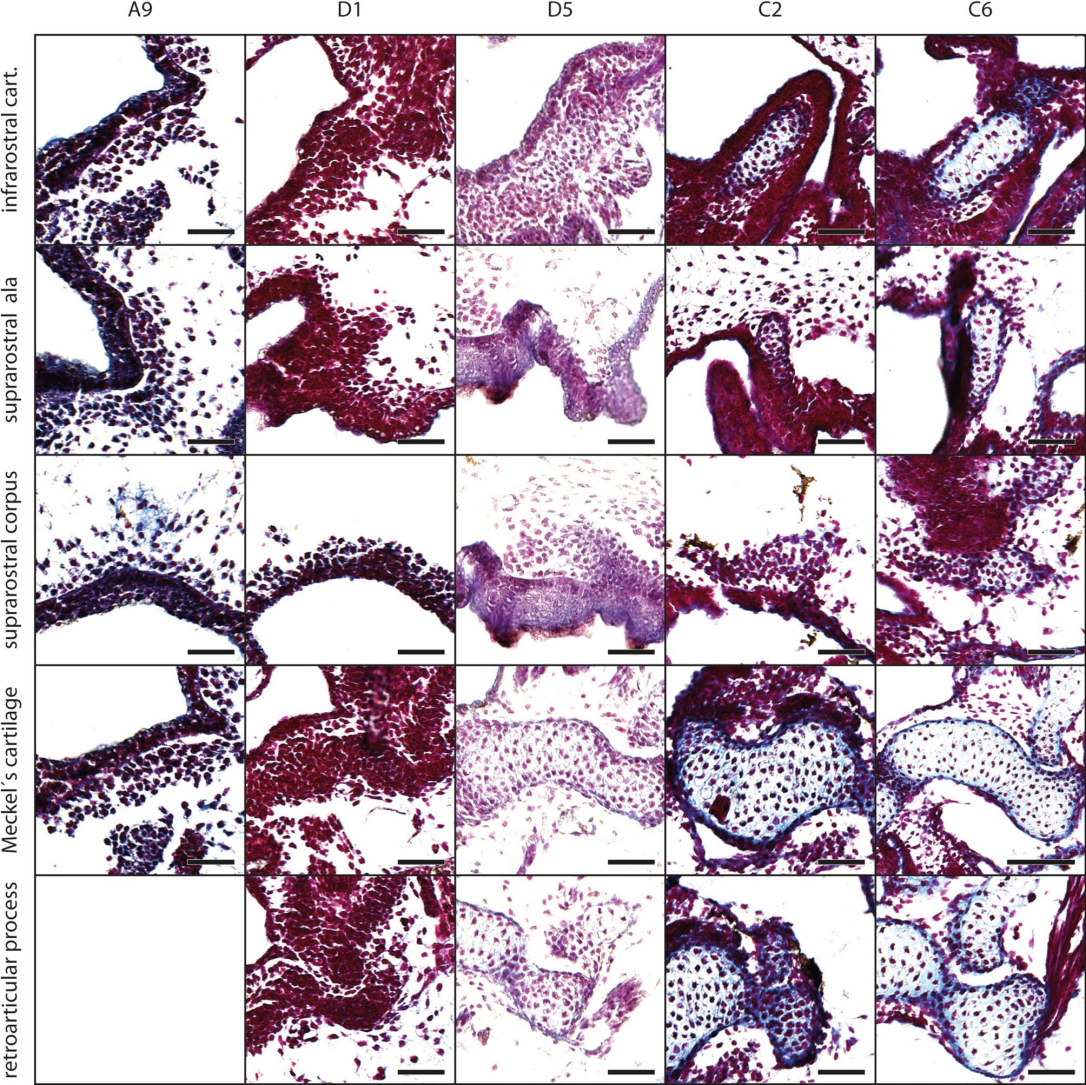
*Smilisca phaeota*, development of mandibular arch-derived cartilaginous structures. Azan‐stained, transverse histological sections of representative stages during development. Stages are arranged in columns from left to right: Stage A9 (A, mesenchymal anlagen), stage D1 (D1, early differentiation into chondroblasts), stage D5 (D5 advanced differentiation stage), stage C2 (C2, early cartilage differentiation), and stage C6 (C6 later cartilage differentiation). The the chondrification process of several cartilages is shown (top to bottom): the infrarostral cartilage (cart.), suprarostral ala, suprarostral corpus, Meckel's cartilage and retroarticular process of Meckel's cartilage. Empty squares indicate the absence of the structure in the respective stage. Scale bars indicate 100 μm.

**Fig. 3:**
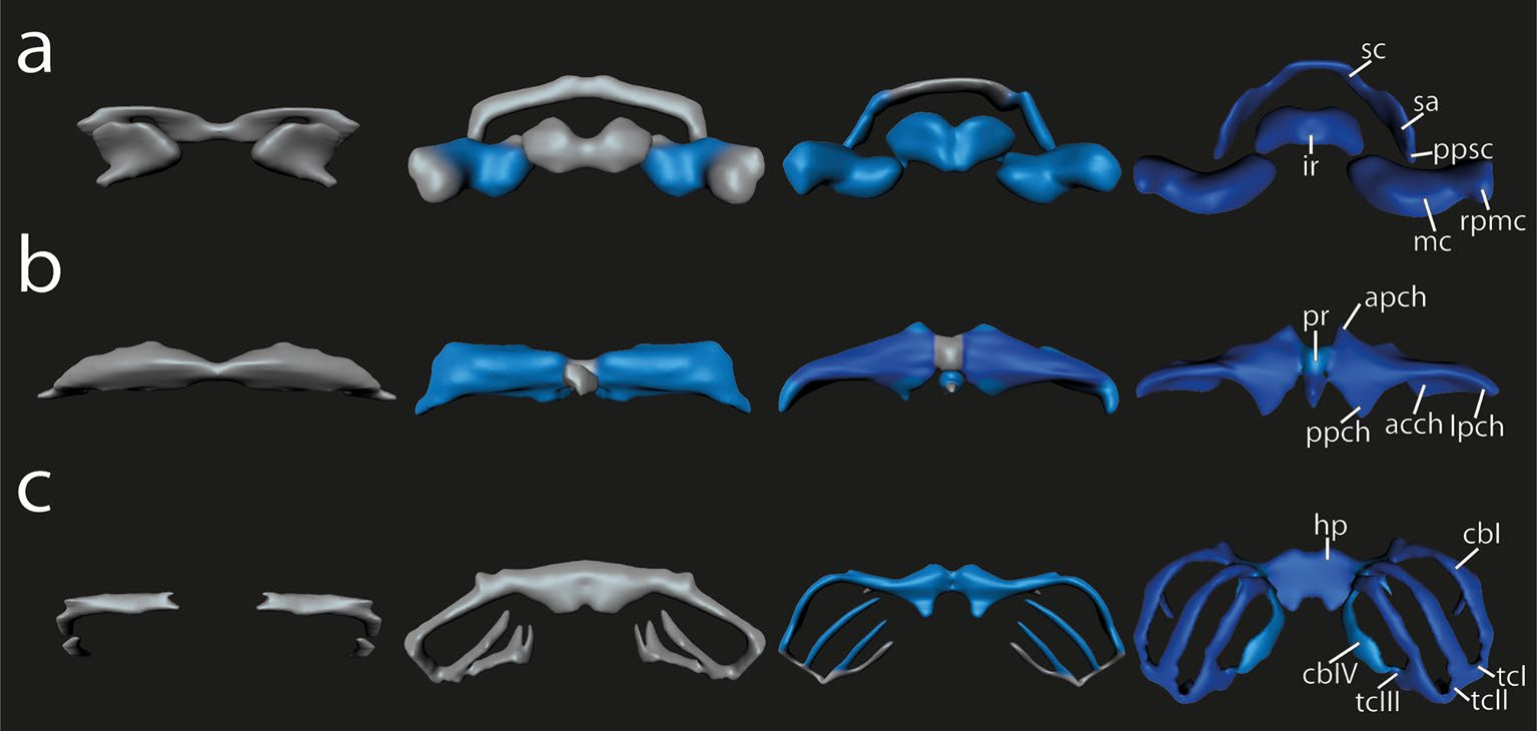
3D reconstructions of the chondrocranium of *Smilisca phaeota* larvae highlighting the cartilaginous development of the (**a**) mandibular arch derivatives, (**b**) hyoid arch derivatives, and (**c**) the derivatives of the branchial arches I–IV. The 3D reconstructions are color‐coded, according to the distinct stage of cartilaginous development: “Light gray” indicates Anlagen are visible as mesenchymal cell clusters. Early chondrification processes are indicated in “light blue” signifying that condensed precartilaginous cell clusters containing chondroblasts are visible. Later differentiation processes resulting in cartilages with distinct chondrocytes and perichondrium are indicated in “dark blue”. Sizes were adjusted. Abbreviations: acch, articular condyle of ceratohyal; apch, anterior process of ceratohyal; cbI‐IV, ceratobranchial I–IV; hp, hypobranchial plate; ir, infrarostral cartilage; lpch, lateral process of ceratohyal; mc, Meckel's cartilage; ppsc, posterior process of suprarostral cartilage; rpmc, retroarticular process of Meckel´s cartilage; sa, suprarostral ala; sc, suprarostral corpus, tcI-III, terminal commissure I–III.

The mesenchymal Anlage of the infrarostral cartilage develops at stage A9 (Fig. 2, for Gosner stages see Table 1) ventromedially to the oral cavity. Laterally the Anlage is limited by the mesenchymal Anlage of Meckel´s cartilage. The Anlage of the infrarostral cartilage extends laterally and increases in height, i.e., the lateral parts are thicker than the median part, until stage A13. At this stage, the intramandibular joint develops at the dorsolateral margin of the mesenchymal Anlage of the infrarostral cartilage. Until stage D5 the Anlage extends obliquely and both infrarostral cartilages form a V in frontal view. At stage D6 chondroblasts appear in the Anlage. The median synchondrosis remains mesenchymal. Later the infrarostral cartilage enlarges obliquely, with the lateral end expanding more dorsally. Chondrocytes appear at stage C5, while the general shape of the cartilage remains the same throughout development (Fig. 3A).

#### Suprarostral cartilage

The mesenchymal Anlagen of both suprarostral alae are visible below the lateral margin of the oral cavity at stage A2. These small spheres reside ventromedially to the Anlagen of the jaw levator muscles (Mm. levatores mandibulae). At stage A4, these spheres begin to enlarge and the mesenchymal Anlage of the suprarostral corpus appears dorsal to the oral cavity as an elongated, thin strip of 1–3 cells thickness (Fig. 3a). During the following stages the thin suprarostral corpus extends laterally and fuses with the Anlagen of the suprarostral alae around stage A8. At stage D5, the mesenchymal Anlage of the posterior process appears on the posterodorsal surface of the suprarostral alae. Chondroblasts develop in the alae at stage D6; they alae more plate-like from this stage onwards. The medial part extends ventrally, whereas the lateral part of the suprarostral corpus extends dorsolaterally and establishes the flat-M-like shape at stage C3. At stage C5, the Anlage of the suprarostral corpus consists of chondroblasts. The chondrocytes appear in the suprarostral alae at stage C7.

#### Meckel’s cartilage

The mesenchymal Anlage appears at stage A1 below the ventrolateral surface of the oral cavity and is horizontally oriented and oval. During further development, the Anlage widens horizontally and is of rod-like shape until stage A12, when the medial part of the cartilage bends ventrally resulting in a convex ventral and a concave dorsal surface. The ventromedial process protrudes slightly from the ventromedial margin and at the laterodorsal margin the retroarticular process develops as a small bulge. From stage D1 onwards, the medial part of the Anlage of Meckel´s cartilage extends anterodorsally and the lateral part posteriorly (Fig. 3a). This process ends at stage D6 when the cartilage acquires its typical sigmoid shape. Chondroblasts appear at stage D2 (body), D4 (retroarticular process), and D6 (ventromedial process). The retroarticular process articulates with the articular process of the palatoquadrate forming the primary jaw joint at stage D6. The Anlage of Meckel´s cartilage widens mediolaterally, and chondrocytes appear at stage C3. At stage C5 the retroarticular process contains chondrocytes. The intramandibular joint is established at stage C7 (Fig. 3a).

#### Palatoquadrate

**Fig. 4:**
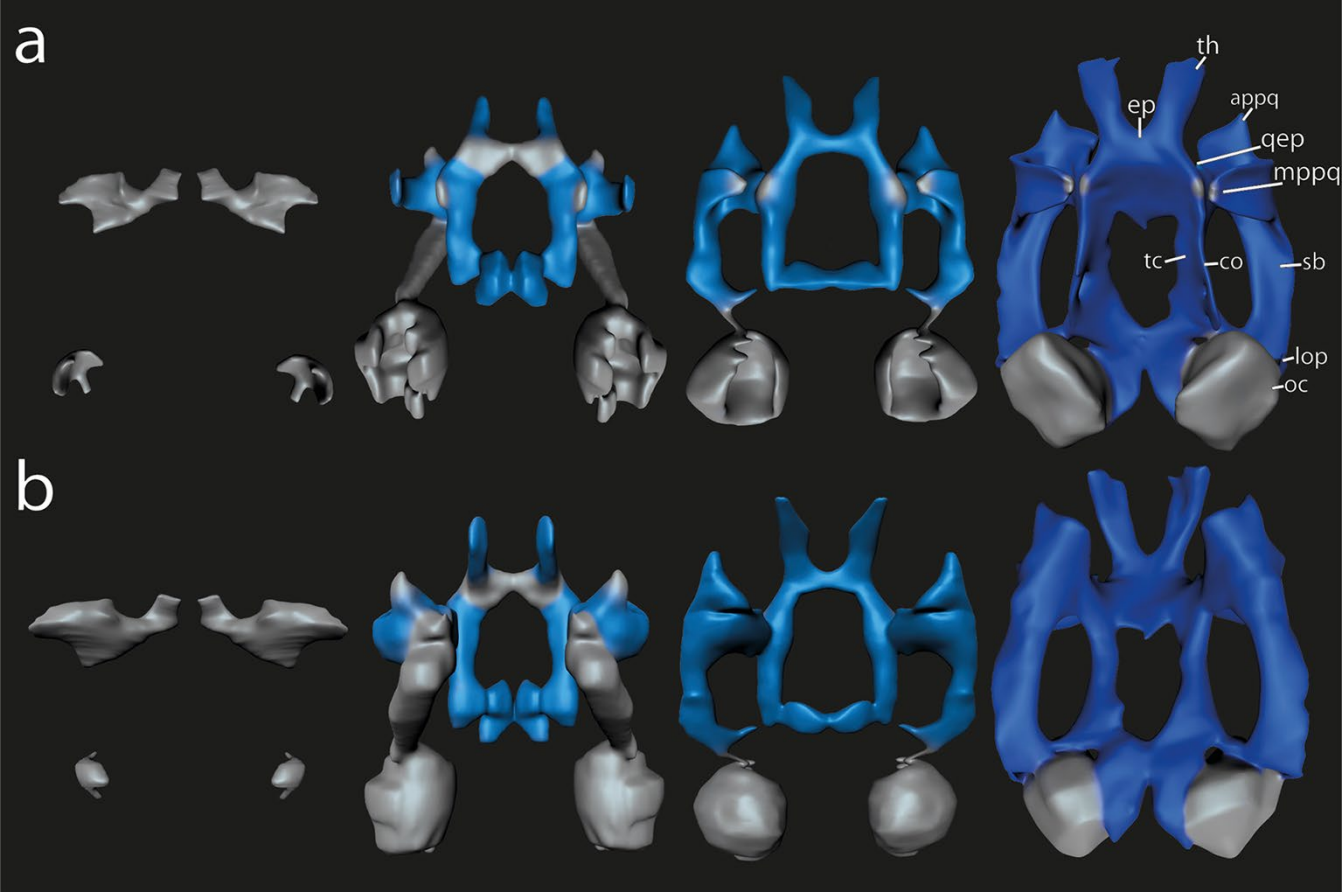
3D reconstructions of the chondrocranium of *Smilisca phaeota* larvae highlighting the cartilaginous development of the neurocranium and palatoquadrate in (**a**) dorsal, and (**b**) ventral view. The 3D reconstructions are color‐coded, according to the distinct stage of cartilaginous development: “Light gray” indicates mesenchymal Anlagen; “light blue” indicates that condensed precartilaginous cell clusters containing chondroblasts, “dark blue” indicates a differentiated e cartilage with chondrocytes surrounded by cytoplasm and bordered by a distinct perichondrium. Sizes were adjusted. Abbreviations: appq, articular process of palatoquadrate; co, orbital cartilage; ep, ethmoid plate; lop, larval otic process; mppq, muscular process of palatoquadrate; oc, otic capsule; qep, quadratoethmoid process; sb, subocular bar; tc, trabecular cartilage; th, trabecular horn.

**Fig. 5:**
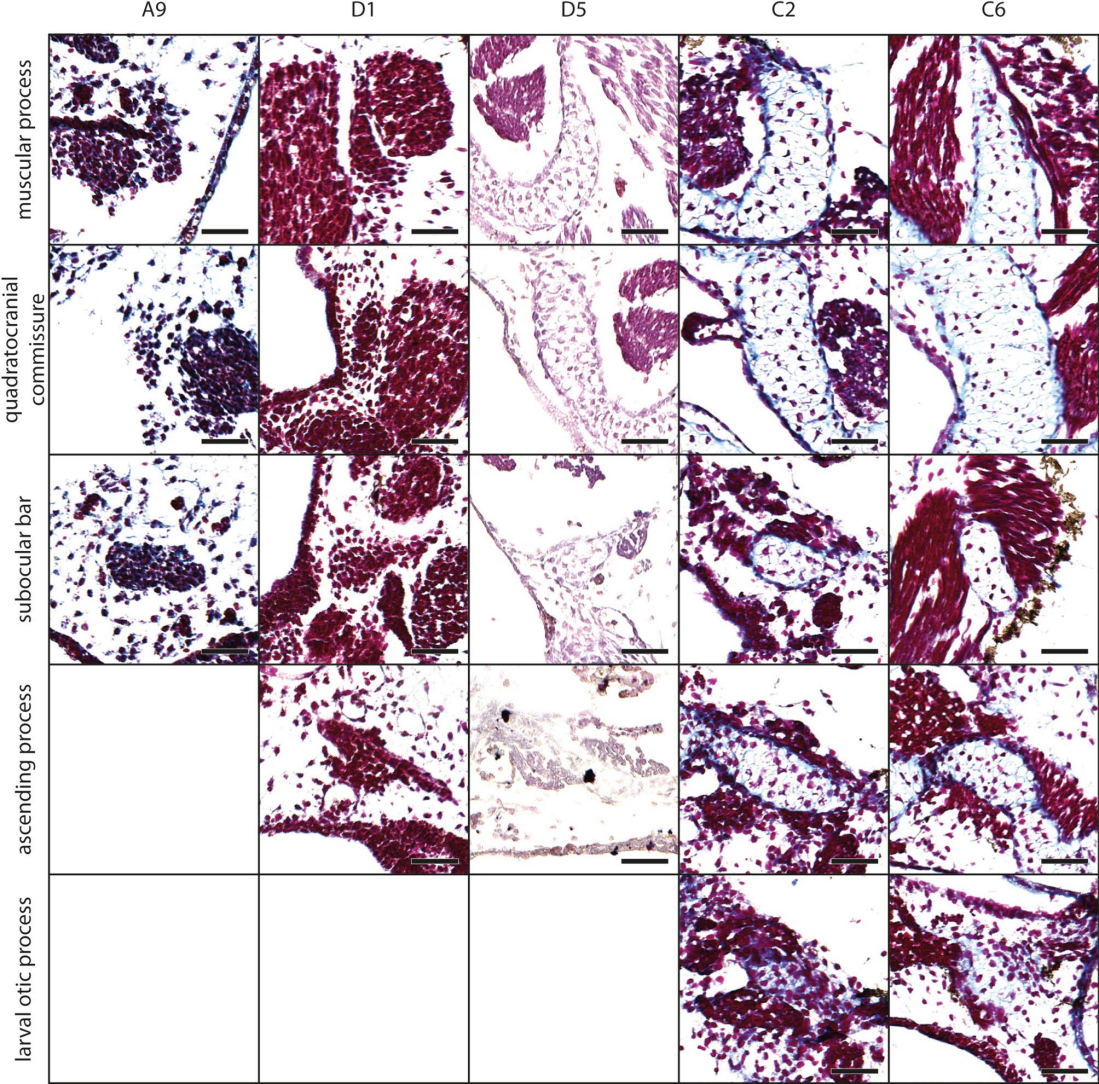
*Smilisca phaeota*, cartilaginous development of the palatoquadrate and its associated structures. Azan‐stained, transverse histological sections of several developmental stages from left to right: stage A9, stage D1, stage D5, stage C2, and stage C6 showing the chondrification process of the muscular process, quadratocranial commissure, subocular bar, ascending process, and larval otic process. See Fig. 2 for more details on the stages. Empty squares indicate the absence of the structure in the respective stage. Scale bars indicate 100 μm.

The mesenchymal Anlage of the subocular bar appears at stage A2, ventral to the eye Anlage and dorsolateral to the oral cavity. The mesenchymal Anlage of the muscular process develops at stage A3 as a small cylindrical condensation lateral to the Anlage of the jaw levator muscles. The Anlage of the subocular bar extends anteriorly and fuses with the mesenchymal Anlage of the muscular process at stage A4 (Fig. 4). At the same stage, the mesenchymal Anlage of the anterior quadratocranial commissure develops medial to the Anlage of the jaw levator muscles. The palatoquadrate is U-shaped in frontal aspect (lateral: muscular process; intermediate: body; medial: anterior quadratocranial commissure) (Fig. 5). At stage A7, at the level of the posterior end of the eye, the mesenchymal Anlage of the hyoquadrate process appears as a small bulge at the ventral surface of the subocular bar. This hyoquadrate process is near the mesenchymal Anlage of the more ventrally located articular condyle of the ceratohyal. The subocular bar extends posteriorly. At stage A10, the ascending process extends as a small rod like cellular condensation dorsomedially on the posterior border of the subocular bar. At stage A12, the mesenchymal Anlage of the articular process appears on the anterior border of the palatoquadrate. Both the quadratocranial commissure and muscular process grow in height and first chondroblasts can be observed at stage D2 (Fig. 4a). Posteriorly, the slender, mesenchymal Anlage of the larval otic process connects to the otic capsule Anlage (D2). At stage D4, chondroblasts appear in the Anlagen of the subocular bar, hyoquadrate process, and ascending process (Fig. 5). Between the dorsal tips of muscular process and quadratocranial commissure develops at stage D5 the mesenchymal Anlage of the quadratoorbital commissure, which does not further differentiate in the stages covered in this study. The muscular process and the anterior quadratocranial commissure are the first Anlagen with chondrocytes (stage C1), followed by the subocular bar (C3), articular and ascending processes (C5), larval otic process (C6), and hyoquadrate process (C7) (Fig. 4a). There is no strict spatial sequence.

#### Ceratohyal

**Fig. 6:**
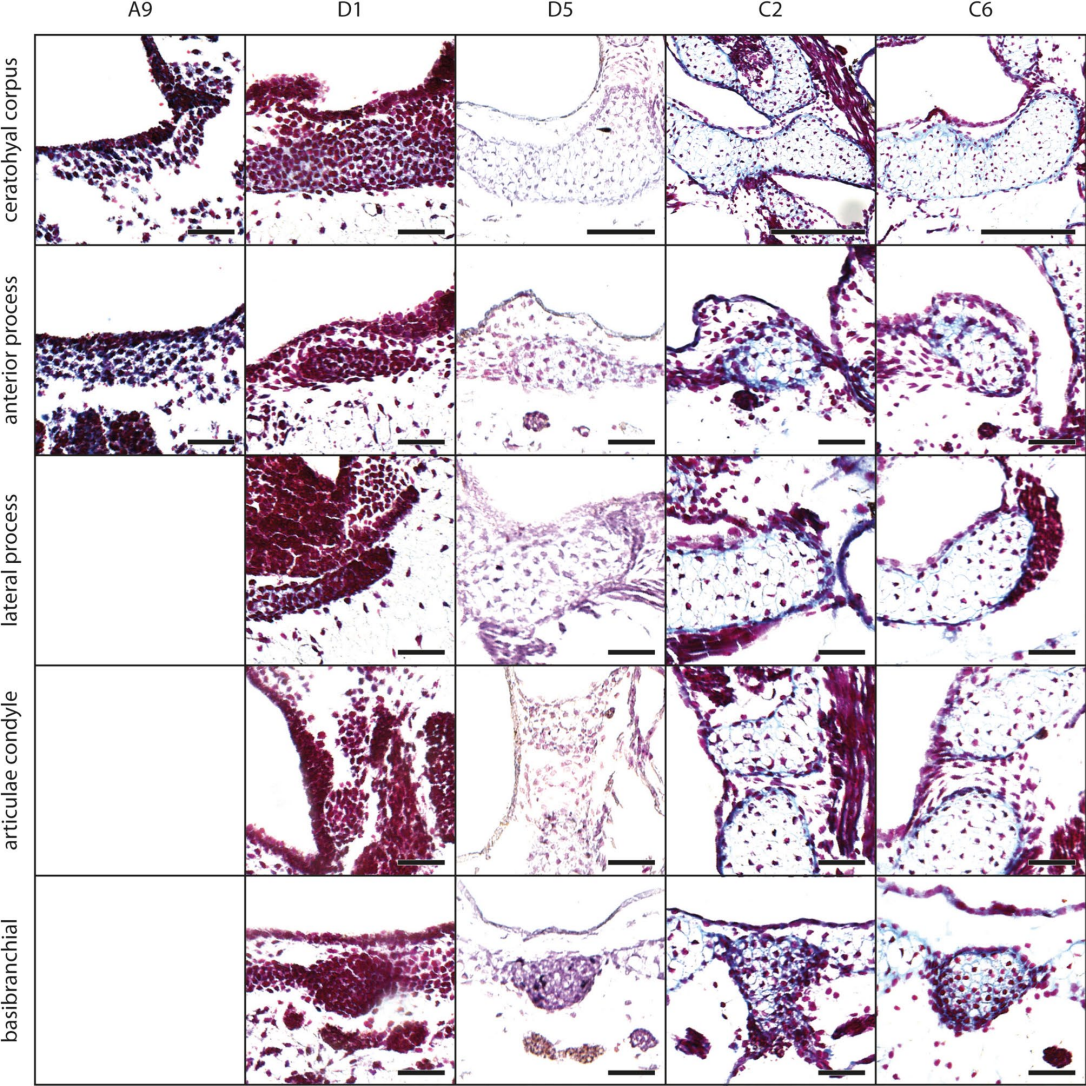
*Smilisca phaeota*, cartilaginous development of the ceratohyal and basibranchial. Azan‐stained, transverse histological sections of stage A9 (first column), stage D1 (second column), stage D5 (third column), stage C2 (fourth column), and stage C6 (fifth column) show the chondrification process of the ceratohyal corpus, anterior process, lateral process, articular condyle, and basibranchial. See Fig. 2 for more details on the stages. Empty squares indicate the absence of the structure in the respective stage. Scale bars indicate 100 μm.

The mesenchymal Anlagen of the ceratohyals appear at stage A2 ventral to the oral cavity. At stage A4, both ceratohyals are connected through the mesenchymal Anlage of the pars reuniens; on the anteromedial surface of the ceratohyal the small mesenchymal Anlage of the anterior process appears. The Anlage of the ceratohyal extends laterodorsally. At stage A7 the mesenchymal Anlagen of the lateral process and the articular condyle develop. The former is bordered by the Anlage of the orbitohyoideus muscle; the latter extends from the mediodorsal surface of the ceratohyal. The mesenchymal Anlage of the posterior process develops as a distinct posteriorly protruding rod from the medial part of the ceratohyal corpus. The previously horizontally oriented lateral process begins to turn vertically at stage A8. At the end of this process (stage D6), the lateral process is nearly at a right angle to the horizontal plane. Chondroblasts can be observed in the corpus and the anterior process at stage D1 (Fig. 6), within the lateral and posterior processes at stage D2 (Fig. 3b), and within the articular condyle at stage D4. Each ceratohyal extends laterally and posteromedially during this phase. At stage C3 the first chondroblasts appear within the pars reuniens, but chondrocytes are not observed in this study. The chondrocytes are present at the stages C1 (ceratohyal corpus), C2 (articular condyle), C3 (anterior and posterior process), and C4 (lateral process) (Figs. 3b, 6).

#### Basibranchial

The oval, mesenchymal Anlage of the basibranchial develops at stage A10, bordered by the mesenchymal Anlagen of the ceratohyals and hypobranchial plate. At stage D1, its anterior margin fuses with the posterior surface of the pars reuniens. At stage D2, the small and caudally bulging mesenchymal Anlage of the urobranchial process appears on the posterior surface of the basibranchial (Fig. 6). The urobranchial process points posteroventrally and remains inconspicuous during the following stages. The first chondroblasts within the basibranchial appear at stage D5 (Fig. 6). The posterior surface of the basibranchial is near the anterior surface of the hypobranchial plate, but both structures do not fuse during the investigated stages. The urobranchial process consists of chondroblasts at stage C3. The basibranchial does not enlarge much during the following stages and it chondrifies at stage C6, followed by the urobranchial process at stage C7.

#### Branchial basket

**Fig. 7:**
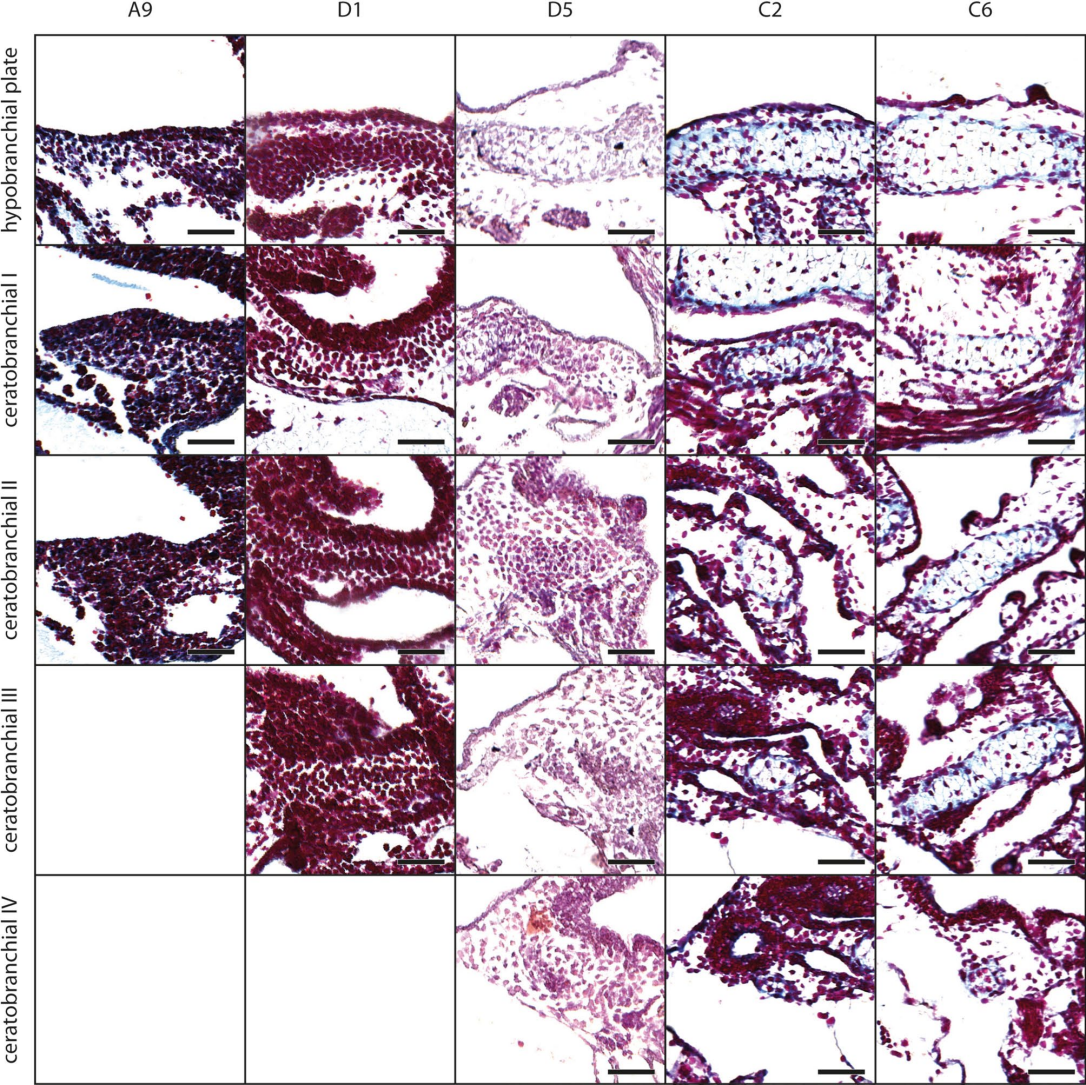
*Smilisca phaeota*, cartilaginous development of the branchial basket. Azan‐stained, transverse histological sections of several developmental stages from left to right: stage A9 (first column), stage D1 (second column), stage D5 (third column), stage C2 (fourth column), and stage C6 (fifth column) showing the chondrification process of the hypobranchial plate and the ceratobranchials I–IV. See Fig. 2 for more details on the stages. Empty squares indicate the absence of the structure in the respective stage. Scale bars indicate 100 μm.

At stage A4, the mesenchymal Anlage of the hypobranchial plate appears below the posteroventral part of the oral cavity, near the Anlagen of the ceratohyals (Fig. 7). The Anlage is short (anteroposterior) but wide (left–right), and fuses with the mesenchymal Anlage of ceratobranchial I at stage D2 (Fig. 7). At stage D3, first chondroblasts differentiate within the hypobranchial plate Anlage and it extends posteriorly. First chondrocytes appear at stage C4. The mesenchymal Anlagen of ceratobranchials I–IV emerge as small horizontally oriented cylindrical rods which grow laterally while their lateral tips bend posteriorly. The ceratobranchial I and II develop almost simultaneously. Ceratobranchial III and IV follow in an anterior to posterior pattern of development, which is also shared by the proximal commissure I-III and the terminal commissure I-III. The mesenchymal Anlagen of ceratobranchial I and II appear within the respective branchial pouches at stage A2. The anterior branchial process develops on the anterior margin of ceratobranchial I at stage D2 (Fig. 3c). Ceratobranchial I consists of chondroblasts at stage D4, and ceratobranchial II at stage D5 (Fig. 7). Ceratobranchials I and II chondrify at stage C5. The mesenchymal Anlagen of ceratobranchials III and IV emerge at stage A8 and D2, respectively. Chondroblasts appear at stage C1 within the Anlagen of ceratobranchial III and anterior branchial process, and at stage C2 within the Anlage of ceratobranchial IV (Figs. 3c, 7). Ceratobranchial III chondrifies at stage C5, anterior branchial process at stage C6, while chondrocytes were not observed within the ceratobranchial IV Anlage in the here investigated specimens. The mesenchymal Anlagen of the terminal commissures appear as a thin cell line between the lateral tips of the respective ceratobranchials at stages A6 (terminal commissure I), A8 (terminal commissure II), and D2 (terminal commissure III). Commissures are wider in older larvae. Terminal commissures I and II consist of chondroblasts at stage C5. A distinct stage where the terminal commissure III consists of chondroblasts was not observed, but at stage C7 it contains chondrocytes (Fig. 3c). The mesenchymal Anlagen of the proximal commissures appear as small stripes connecting the proximal parts of the respective ceratobranchials at stages C3 (proximal commissure I), C5 (proximal commissure II), and C6 (proximal commissure III). All proximal commissures lack stages with clearly visible chondroblasts but are chondrified at stage C7.

#### Neurocranium

**Fig. 8:**
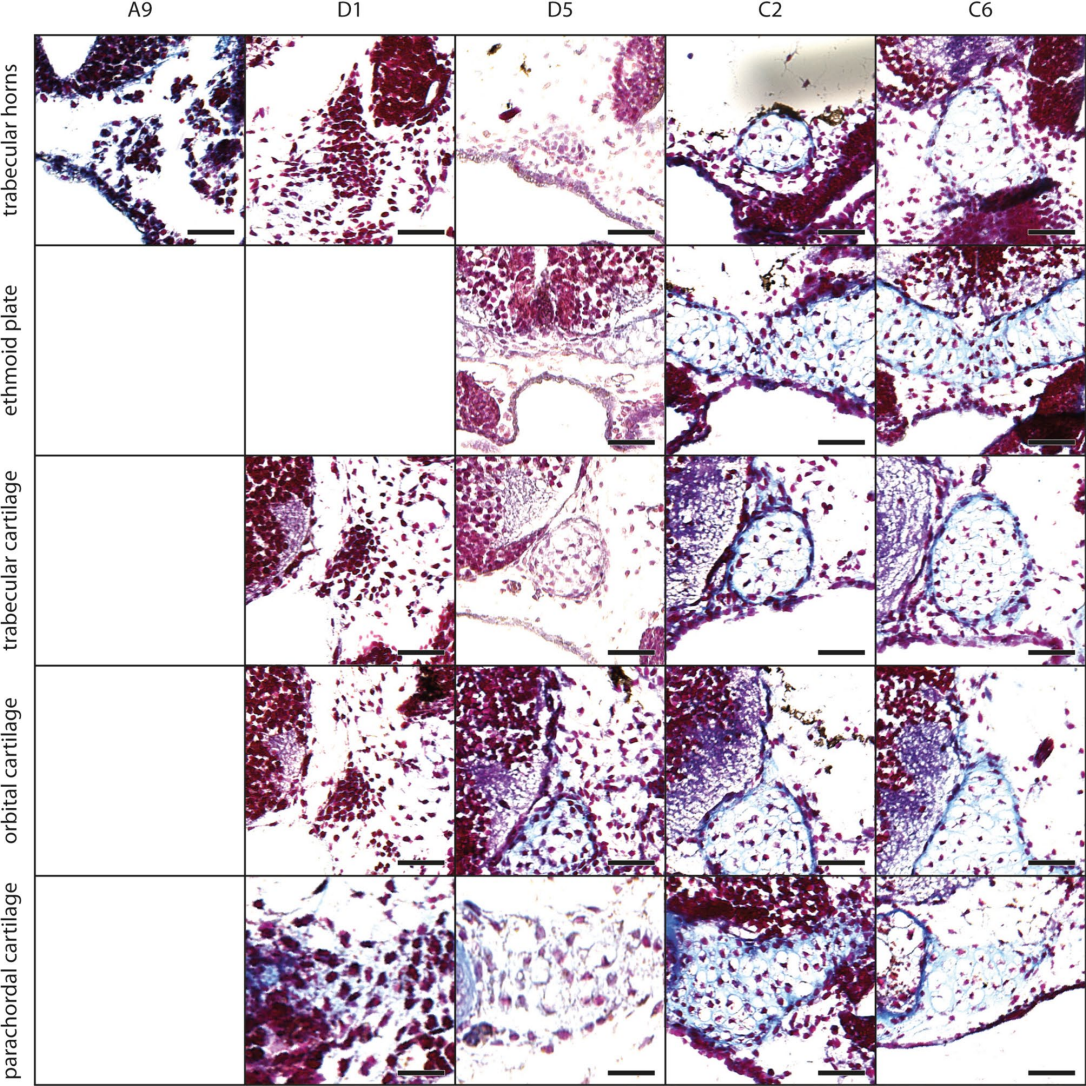
*Smilisca phaeota*, cartilaginous development of the neurocranium. Azan‐stained, transverse histological sections of several developmental stages from left to right: stage A9 (first column), stage D1 (second column), stage D5 (third column), stage C2 (fourth column), and stage C6 (fifth column) showing the chondrification process of the trabecular horns, ethmoid plate, trabecular cartilage, orbital cartilage, and parachordal cartilage. See Fig. 2 for more details on the stages. Empty squares indicate the absence of the structure in the respective stage. Scale bars indicate 100 μm.

The mesenchymal Anlagen of the otic capsules and the trabecular horns develop posterolaterally (stage A2) and posterodorsally (stage A3), respectively, to the suprarostral alae. The Anlagen of the trabecular horns elongate dorsally and caudally. At stage A5, the mesenchymal Anlage of the trabecular cartilage emerges from the posterior surface of the trabecular horn and extends posteriorly. The mesenchymal Anlagen of the parachordal cartilages develop at stage A10 between the anterior margin of both otic capsules. At stage A11, the thin mesenchymal Anlage of the ethmoid plate connects the posterior ends of the trabecular horns. At stage D1, the mesenchymal Anlage of the orbital cartilage arises from the dorsal surface of the trabecular cartilage as a thin, dorsally extending band of cells (Fig. 8). At the same stage, the anterolateral surface of the trabecular cartilage fuses with the Anlage of the quadratocranial commissure. Simultaneously, the posterior part of the trabecular cartilage fuses with the Anlage of the parachordal cartilage. The Anlagen of the quadratocranial commissure, the trabecular horn, and the parachordal cartilage consist of chondroblasts at stage D2 (Fig. 4), followed by the Anlagen of the orbital cartilage at stage D3. During further development the orbital cartilage extends dorsally, the ethmoid plate becomes thicker and extends posteriorly, and the parachordal cartilage extends medially, which results in a plate-like posteroventral neurocranium at stage D6. The chondrification of the neurocranial parts occurs from anterior to posterior. It starts at stage C2 within the trabecular horns, ethmoid plate, and trabecular cartilages (Fig. 8), followed by the orbital cartilage (C6) and the parachordal cartilage (C7; Fig. 4). The Anlagen of the otic capsules remain mesenchymal during the investigated stages.

## Discussion

A growing number of studies are focusing on the sequential description of the chondrocranial development of vertebrates. The comparison among different clades enables the identification of evolutionary trends, clade defining features, and the reconstruction of the condition in the last common ancestor of all vertebrates. The sequence of chondrocranial development is available for several chondrichthyans^23,24^, osteichthyans^23,25,26^, the coelacanth (*Latimeria sp.)*^27^; *Neoceratodus forsteri*^28^, different reptiles (Lepidosauria)^29–33^, Testudines^34,35^, Crocodilia^36,37^, and birds (Aves)^38^. General trends within vertebrates were proposed: (I) anteroposterior direction of viscerocranial development; (II) posteroanterior direction of neurocranial development; (III) first structures to develop are otic capsules, parachordals, and initially paired trabecular cartilages; and (IV) neurocranial development starts before viscerocranial development. However, anuran species deviate from all four postulated general patterns.

The anteroposterior direction of viscerocranial development is thought to be the ancestral condition and was repeatedly described within different non-anuran vertebrates^23–25^. Recently, the viscerocranial development of anurans was described as mosaic-like^15–18^. In anurans, complex and species-specific patterns exist, where, in most cases, parts of the ceratohyal, a derivative of the second pharyngeal arch (PA2), develop before derivatives of PA1. Furthermore, the anuran specific larval structures infrarostral and suprarostral cartilages, which are derived from PA1, develop after structures of PAs2-5. Comparing the onset of chondrification of different viscerocranial structures (whole cartilages, no processi for the sake of readability) in *Smilisca phaeota*, we observe the following sequence: first palatoquadrate (PA1) and ceratohyal (PA2); followed by Meckel´s cartilage (PA1); third infrarostral cartilage (PA1), ceratobranchial I (PA3), ceratobranchial II (PA4), and ceratobranchial III (PA5); fourth suprarostral cartilage (PA1); and fifth ceratobranchial IV (PA6). Even if we exclude the anuran unique features (infrarostral and suprarostral cartilage) the pattern is not anteroposterior sequential, which means that the evolution of these novelties cannot be the only explanation for the deviation from the ancestral pattern. Non-anteroposterior patterns were also described in other anurans^15–18^. This supports the scenario, that the anuran viscerocranial chondrification sequence is more flexible than in non-anuran vertebrates. The reason for this flexibility remains unknown.

A posteroanterior pattern of neurocranial development, and the development of parachordals, otic capsules, and trabecular cartilages as first neurocranial structures, can be observed in a variety of non-anuran species^25,27,28,32,35–37^. *S. phaeota* and other anurans^15–18^ deviate from this pattern. Posterior cartilages of the neurocranium like the parachordals grow anteriorly and anterior cartilages such as the trabecular horns grow posteriorly. The respective neurocranial parts fuse in later stages. Deviations from the posteroanterior pattern were also observed in the turtle *Caretta caretta*^34^ and in the squamate *Ptychoglossus bicolor*^31^. The latter exhibits a pattern similar to anurans with the trabecular cartilages not growing out of the parachordals in anterior direction, but they emerge anteriorly and grow posteriorly to fuse later with the parachordals. The early development of the trabecular horns in all anurans investigated so far is another deviation of the ancestral pattern^15–18,39^. The development of the parachordals and the trabecular cartilages are clearly delayed in *S. phaeota* compared to other vertebrate species^25,33^ where these structures mark the beginning of viscerocranial chondrification.

The general patterns of anuran chondrocranial development were discussed before. A summary of previous studies^14,16–18,29,40–44^ has led to the formulation of hypothetical characteristics of cartilaginous development in different anuran families^15^. Here, we compare the features of *S. phaeota’*s development to that of other anurans. Certain features of anuran cartilaginous development are shared by *S. phaeota,* including the anterior to posterior sequence of the development of the palatoquadrate-anchoring processes (quadratocranial commissure, ascending process, larval otic process), and the late development of the synoptic tectum (pers. obs. of later stages not covered here).

Anurans contain two clades, Leiopelmatidae (including Ascaphidae) and Lalagobatrachia (all other frogs)^45^. Treating *Ascaphus* as an outgroup and comparing it to the six species investigated by us (Table 2), one can speculate about phylogenetic relevant characteristics. The sequence of chondrocranial development of *S. phaeota* supports several suggested lalagobatrachian features.

1. The development of the suprarostral alae before the suprarostral corpus was observed in all species investigated so far, except in *A. truei* and *X. laevis,* where the suprarostral corpus develops before the alae^14–16^. The possible scenarios for the ancestral sequence of suprarostral development in anurans were discussed before^15^ and the development in *S. phaeota* does not provide further resolution.
2. The development of *S. phaeota* partially supports other lalagobatrachian features, such as the chondrification of the PA1-derived infrarostral cartilage after the PA3-derived ceratobranchial I, and the muscular process being the first structure which chondrifies within the palatoquadrate. Considering that the quadratocranial commissure chondrifies at the same stage, it is questionable whether this condition can be accurately classified as a defining feature of lalagobatrachians.
3. The ceratohyal corpus is among the first structure which consists of chondroblasts and chondrocytes in *S. phaeota*, which further supports the hypothesis that the temporal displacement of ceratohyal development is an important feature at least for the lalagobatrachian clade^15,18,39^ (because data from ascaphids and leiopelmatids is currently missing).

**Table 2:** Studied species versus diverse characters.

Species	Characters as larvae	Suborder	Family,	References
*Xenopus laevis*	Filter feeder, free water column, close to surface	Mesobatrachia	Pipidae	^16,56^
*Hymenochirus boettgeri*	Miniaturized predator with highly modified jaw apparatus, free water column			^39^
*Discoglossus scovazzi*	Scraping suspension feeders, close to ground	Archeobatrachia	Discoglossidae	^18^
*Bombina orientalis*			Bombinatoridae	^17^
*Bufo bufo*		Neobatrachia	Bufonidae	^15^
*Smilisca phaeota*			Hylidae	Present study

All are metamorphosing species, all are aquatic as larvae.

Over the past years, the chondrocranial development in several anuran species was described (Table 2). While there are some similarities, there are many differences within anurans but also between anurans and the developmental sequence of other vertebrates. This is partially due to the resolution of developmental series, how studies group elements, what is defined as first appearance, or when the scoring is compared (e.g., chondroblast appearance vs. chondrocyte appearance).

Notably, so far, no comparison revealed a strong phylogenetic signal for sequence of chondrocranium nor head muscle development. The previous paragraphs regarding potential apomorphic traits in Lalagobatrachia are highly speculative considering the low number of species studied and that the outgroup (*A. truei*) was investigated with different methods. In fact, head muscle development has so many heterochronies (changes in the timing of first appearance of a character compared to another character) that muscles could only be compared in groups of PA-derivatives^46,47^. Therefore, the specific developmental sequence is less restricted than thought and might be related to lifestyle adaptations (feeding, borrowing, habitat, etc.). With respect to head muscle development in amphibians, no such connection could be made^47^. But with the growing number of sequences available for the chondrocranium development in anurans a fresh look into the correlation of any adaptation and sequence might be useful.

Most tadpoles are specialized herbivores, while there are also a few predatory tadpoles. There are three main ways of herbivorous feeding among tadpoles. They are feeding on planktonic material in the water column (filter feeders), detritus in pond sediment, or scraping material from submerged substrates such as rocks and reeds^48^. The six anuran species studied so far (Table 2) include highly specialized tadpoles, like the miniaturized, predatory suction feeder *Hymenochirus boettgeri*, and generalized tadpoles (e.g., *Bufo bufo*) that are scraping suspension feeders or that filter feed (*Xenopus laevis*). Four species in Table 2 have the same feeding style and it could be expected that these share a more similar developmental sequence than compared to the other two species.

We have compared the sequences of development by first appearance of representative elements of the respective PAs (Table 3) without being able to identify any pattern related to any lifestyle. The mandibular arch (PA1) is even inconsistent in the order in which dorsal (palatoquadrate related) or ventral (Meckel’s cartilage related) elements show first a specific developmental stage. While the branchial arches (BAs1–4 = PAs3–6) develop mostly from anterior to posterior, the BA2 element develops early with respect to the first BA1 element in *H. boettgeri* and during chondroblast condensation in *B. bufo*. The neurocranial elements differentiate (black in Table 3) late in *X. laevis* (with BA2/PA4 elements) and *Bombina orientalis* (with BA1/PA3 elements) as compared to other species studied so far, where they usually differentiate with 1st or 2nd PA elements. Unfortunately, these are mere interesting observations, without providing insights regarding evolutionary trajectories because deriving features for a large clade from the comparison of only six representatives is challenging and, as expected, some previously assumed properties do not hold up to comparison^15^. However, it is necessary to lay the foundations for future research, in which hypotheses can be confirmed, modified, or refuted.

**Table 3:**
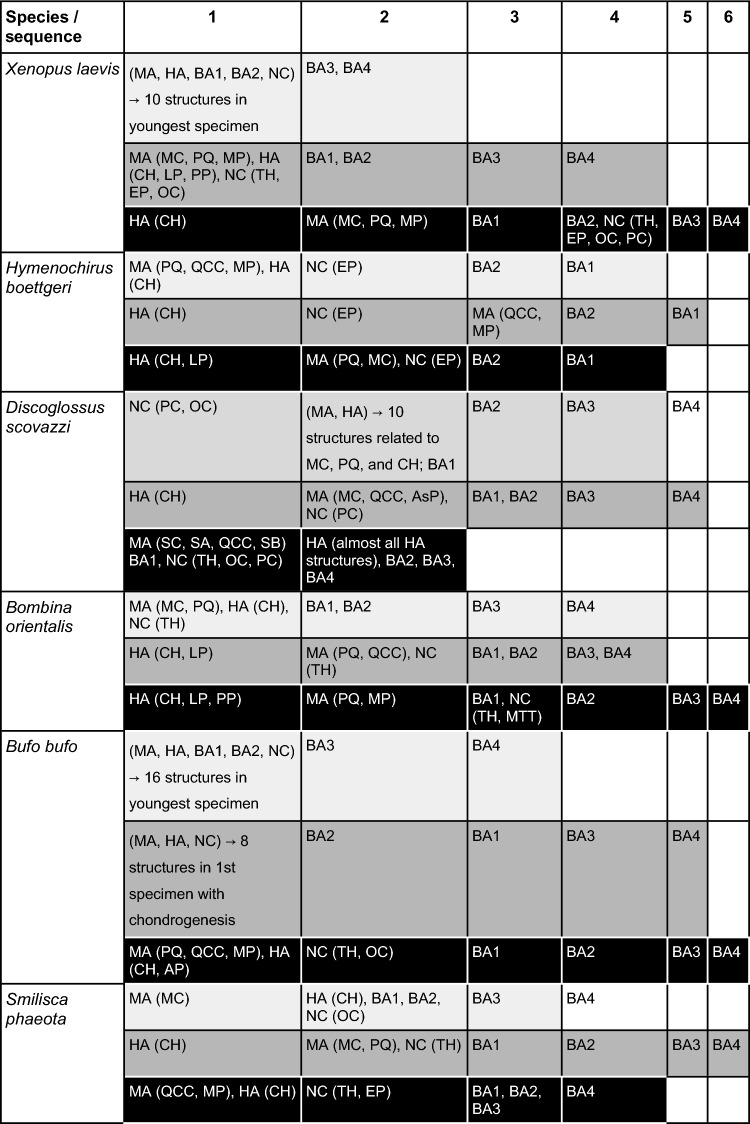
Sequence of development of pharyngeal arches (PAs) 1–6 and Neurocranium (NC).

Structures in the same box represent simultaneous appearances. The branchial arches (BAs) are marked by the development of the ceratobranchialia, the mandibular (MA) and hyoid arch (HA) are marked by the first appearance of any part of the arch. Gray scale: (light gray) mesenchymal structures are visible as condensed cell clusters; (middle gray) chondroblasts form condensed precartilaginous cell clusters; and (black) chondrocytes rich in cytoplasm and bordered by a perichondrium. Abbreviations: MA—1st PA = Mandibular arch, HA—2nd PA = Hyoid arch, BA1 (Branchial arch 1)—3rd PA (Pharyngeal arch), BA2—4th PA, BA3—5th PA, BA4—6th PA, NC—Neurocranium.

Abbreviations related to the arches:

MA: MC—Meckel’s cartilage, PQ—palatoquadrate, QCC—quadratocranial commissure, MP—muscular process, AsP—ascending process, SC—suprarostral cartilage, SA—suprarostral alae, SB—subocular bar.

HA: CH—ceratohyal, LP—lateral process, AP—anterior process, PP—posterior process.

NC: OT—otic capsule, TH—trabecular horns, EP- ethmoid plate, PC—parachordal cartilage, MTT—marginal tectal taenia.

The chondrocranial development of *S. phaeota* supports only one feature, which was postulated before as defining for the neobatrachians, which is the delayed basibranchial development in comparison to the palatoquadrate development. This feature is a deviation from the proposed lalagobatrachian pattern, where both structures develop simultaneously. Other deviations like the asynchronous development of the articular condyle of the ceratohyal and the hyoquadrate process of the palatoquadrate, and a flexible pattern of ceratobranchial development are found in *S. phaeota* and in *B. bufo*^15^. Which trends are present within neobatrachians regarding these features needs further investigation.

The appearance of the parachordal cartilage marks the beginning of chondrocranial development in many non-anuran vertebrates^25–27,32,35–37^. In most anurans, the parachordal cartilage is the first neurocranial derivative to develop, but in *S. phaeota* it is so delayed that we could not observe it in this study. The deviation of this pattern may be a feature unique to *S. phaeota* or indicates that also the neurocranial development is much more flexible than previously thought.

## Conclusions

1. The anuran-specific features observed in *Smilisca phaeota* as well in other anuran lavae challenge the proposed general trends within vertebrates. This flexibility in viscerocranial development in anurans suggests unique evolutionary or functional adaptations.
2. *S phaeota* and other anurans deviate also in the neurocranial patterning, further emphasizing the variability in anuran chondrocranial development. This challenges the proposed uniform neurocranial development sequence across vertebrates.
3. *S. phaeota* shares certain characteristics with other anuran families, supporting proposed features within the Lalagobatrachia clade: the development of the suprarostral alae before the suprarostral corpus, the chondrification of the infrarostral cartilage after ceratobranchial I, and the acceleration of ceratohyal development. However, the specific sequence of chondrocranial development in anurans may not exhibit a strong phylogenetic signal, and it is likely influenced by lifestyle adaptations with or without reduced developmental constraints.
4. Our study emphasizes the complexity of developmental processes and highlights the need for additional research to explore potential correlations between adaptation, lifestyle, and chondrocranial development.

In summary, the chondrocranial development of *S. phaeota* provides novel insights into the flexibility and adaptability of anuran chondrocranial patterns, challenging established norms and paving the way for further investigations into the intricate interplay between evolution, adaptation, and developmental sequences in vertebrates.

## Methods

### Husbandry and staging

Fertilized eggs of Smilisca p*haeota* were provided by a private breeder. Clutches of different origin were transferred to petri dishes and cultured in 0.1X modified Barth's saline^49^ at temperatures between 20 and 27 °C to collect an uninterrupted series of stages. All specimens (embryos and larvae) were staged according to the simplified staging table for anuran embryos and larvae by Gosner^50^ and designated as “Go”. A developmental series was established by collecting embryos and larvae from defined stages between Go 19 and Go 35 (n = 123). Anesthesia was performed with 1% tricaine methanesulfonate (MS-222) according to the animal welfare protocols of the Friedrich-Schiller-University Jena, Germany. Specimens were fixed in 4% phosphate-buffered formalin (PFA). Histological sections and cleared-and-stained larvae are stored at the Institute of Zoology and Evolutionary Research, Friedrich-Schiller-University, Jena, Germany.

### Tissue staining

The PFA-fixed specimens were dehydrated, embedded in paraffin, and serial sectioned at 7 μm thickness using a rotary microtome (Microm, HM 355 S). Sections were stained using the Heidenhain Azan technique^51^. Images were captured with a Hitachi HV-F202SCL camera mounted on a Zeiss AxioScanZ.1 microscope operated with Zen 3.1 software. The clearing-and-staining procedure followed the protocol of Dingerkus and Uhler^52^ except that alizarin red was not used as this would stain bones which are absent in the Gosner stages analyzed here (Go 19–26, Table 1). Cleared-and-stained specimens were examined with a Zeiss Stemi 11 and images were taken with an attached camera (ColorView) operated by AnalySIS software. For μCT-scanning, specimens were dehydrated in a graded series of ethanol to 100% (one day for each step). After dehydration, the samples were stained with an alcoholic iodine solution (1%) for two weeks. Prior to scanning they were rinsed in absolute ethanol and mounted in Falcon tubes to prevent movement during scanning.

### µCT

Scans of the larval skull were acquired using a SkyScan221 μCT scanner (Bruker, Belgium) at the Max-Planck-Institut für Menschheitsgeschichte Jena, Germany. A beam strength of 60 kV and 250 μA was used. A 360° scan with 0,180° rotation steps and an exposure time of 260 ms was chosen (pixel size 5,59 µm).

### Image processing

The digitized images (tagged image file format, TIFF) of the respective histological sections were captured and later exported to Fiji software^53^. The sectional images of each individual were stacked and aligned using first the least squares (rigid) and second the elastic nonlinear block correspondence mode from theTrakEM2 plugin for Fiji^54^. The resulting aligned stacks were exported in TIFF. Skeletal structures were segmented using Amira 6.0.1. 3D analysis software (FEI Visualization Sciences Group). TIFF files from the µCt were imported directly into Amira without the additional step in Fiji. Polygonal surfaces were rendered and then exported to Wavefront OBJ file format for further processing in Autodesk Maya 2023 (Autodesk, Inc.). The surfaces were smoothed, the number of polygons reduced, and the surfaces arranged. Autodesk Mudbox 2023 (Autodesk, Inc.) was used for the final composition, coloring and rendering of the images. All images were edited and arranged using Adobe Photoshop CS6 and Adobe Illustrator CS6 (Adobe Inc.).

### Scoring

We evaluated 81 cartilaginous structures defined in our previous work^18^ from 76 specimens. A total of 45 out of 81 characters were identified in *S. phaeota*. The terminology used here follows the guidelines introduced by Haas^55^ for cartilaginous characters (anglicized terms). The following four distinct states of cartilaginous development were scored: (1) cartilaginous structures are absent; (2) mesenchymal structures are visible as condensed cell clusters; (3) chondroblasts form condensed precartilaginous cell clusters with clearly visible nuclei; and (4) chondrocytes rich in cytoplasm and bordered by a clearly visible perichondrium are present. All structures were scored according to the first appearance principle. If a particular state was visible within a structure, the entire structure was scored in that state, even if parts of the structure remained in an earlier state. Additionally, we used the previously defined three different stages of development^18^. Each stage is defined by a specific combination of cartilaginous structures in different cartilaginous developmental states. A1 (Anlage 1) starts with the appearance of the first mesenchymal Anlage, D1 (Differentiation 1) starts with the appearance of the first Anlage containing chondroblasts, and stage C1 (Cartilage 1) starts with the appearance of the first chondrocytes within the respective structure. The following enumeration of stages (e.g. A1, A2, A3…; D1, D2, D3…; C1, C2, C3…) is the result of additional cartilaginous structures entering different states. Notably, since the staging dependent of the developmental progress of the chondrocranium is more detailed than the Gosner stages, several stages of the A, D, or C stages can be included in one Gosner stage.

## Data Availability

The datasets used and/or analysed during the current study are available from the corresponding author (PL) on reasonable request.

## References

[1] Oisi, Y., Ota, K. G., Fujimoto, S. & Kuratani, S. Development of the chondrocranium in hagfishes, with special reference to the early evolution of vertebrates. *Zool. Sci.* **30**, 944–961. 10.2108/zsj.30.944 (2013). 10.2108/zsj.30.944 24199860

[2] Kaucka, M. & Adameyko, I. Evolution and development of the cartilaginous skull: From a lancelet towards a human face. *Semin. Cell Dev. Biol.*10.1016/j.semcdb.2017.12.007 (2017). 29248472 10.1016/j.semcdb.2017.12.007

[3] Fish, J. L. Evolvability of the vertebrate craniofacial skeleton. *Semin. Cell Dev. Biol.* **91**, 13–22. 10.1016/j.semcdb.2017.12.004 (2019). 29248471 10.1016/j.semcdb.2017.12.004 PMC5999547

[4] Gans, C. & Northcutt, R. G. Neural crest and the origin of vertebrates: A new head. *Science* **220**, 268–273 (1983). 17732898 10.1126/science.220.4594.268

[5] Schultze, H.-P., Hanken, J. & Hall, B. Patterns of diversity in the skulls of jawed fishes. *The skull* **2**, 189–254 (1993).

[6] Depew, M. J. & Simpson, C. A. 21st century neontology and the comparative development of the vertebrate skull. *Dev Dyn* **235**, 1256–1291. 10.1002/dvdy.20796 (2006). 16598716 10.1002/dvdy.20796

[7] Schoch, R. R. Skull ontogeny: Developmental patterns of fishes conserved across major tetrapod clades. *Evol. Dev.* **8**, 524–536 (2006). 17073936 10.1111/j.1525-142X.2006.00125.x

[8] Piekarski, N., Gross, J. B. & Hanken, J. Evolutionary innovation and conservation in the embryonic derivation of the vertebrate skull. *Nat Commun* **5**, 5661. 10.1038/ncomms6661 (2014). 25434971 10.1038/ncomms6661 PMC4251486

[9] Amano, O. *et al.* Meckel’s Cartilage: Discovery, embryology and evolution:—Overview of the specificity of Meckel’s Cartilage. *J. Oral Biosci.* **52**, 125–135 (2010).

[10] Kuratani, S., Matsuo, I. & Aizawa, S. Developmental patterning and evolution of the mammalian viscerocranium: genetic insights into comparative morphology. *Dev Dyn* **209**, 139–155 (1997). 9186050 10.1002/(SICI)1097-0177(199706)209:2<139::AID-AJA1>3.0.CO;2-J

[11] Kuratani, S. The neural crest and origin of the neurocranium in vertebrates. *Genesis* **56**, e23213 (2018). 30134067 10.1002/dvg.23213

[12] Frost, D. R. & Darrel, R. Amphibian Species of the World: an Online Reference. Version 6.2. Electronic Database accessible at https://amphibiansoftheworld.amnh.org/index.php (2024).

[13] Ford, L. S. & Cannatella, D. C. The major clades of frogs. *Herpetol. Monogr.* **7**, 94–117 (1993). 10.2307/1466954

[14] Reiss, J. O. Early development of chondrocranium in the tailed frog *Ascaphus truei* Amphibia: Anura): Implications for Anuran palatoquadrate homologies. *J. Morphol.* **231**, 63–100 (1997). 8946738 10.1002/(SICI)1097-4687(199701)231:1<63::AID-JMOR6>3.0.CO;2-P

[15] Lukas, P. Embryonic pattern of cartilaginous head development in the European toad, Bufo bufo. *J. Exp. Zool. Part B Mol. Dev. Evol. *(2023). 10.1002/jez.b.23214 37358281

[16] Lukas, P. & Olsson, L. Sequence and timing of early cranial skeletal development in *Xenopus laevis*. *J. Morphol.* **279**, 62–74 (2018). 28960402 10.1002/jmor.20754

[17] Lukas, P. & Olsson, L. Sequence of chondrocranial development in the oriental fire bellied toad *Bombina orientalis*. *J. Morphol.* **281**, 688–701. 10.1002/jmor.21138 (2020). 32383540 10.1002/jmor.21138

[18] Lukas, P. & Ziermann, J. M. Sequence of chondrocranial development in basal anurans—Let’s make a cranium. *Front. Zool.* **19**, 17 (2022). 35505372 10.1186/s12983-022-00462-z PMC9066780

[19] Darst, C. R. & Cannatella, D. C. Novel relationships among hyloid frogs inferred from 12S and 16S mitochondrial DNA sequences. *Mol. Phylogenet. Evol.* **31**, 462–475 (2004). 15062788 10.1016/j.ympev.2003.09.003

[20] Faivovich, J. *et al.* Systematic review of the frog family Hylidae, with special reference to Hylinae: Phylogenetic analysis and taxonomic revision. *Bull. Am. Museum Nat. Hist.* **2005**, 1–240 (2005).

[21] Duellman, W. E. The hylid frogs of Middle America. (1970).

[22] Wiens, J. J., Fetzner, J. W. Jr., Parkinson, C. L. & Reeder, T. W. Hylid frog phylogeny and sampling strategies for speciose clades. *Syst. Biol.* **54**, 778–807 (2005). 16243760 10.1080/10635150500234625

[23] Gillis, J., Modrell, M. & Baker, C. A timeline of pharyngeal endoskeletal condensation and differentiation in the shark, *Scyliorhinus canicula*, and the paddlefish, *Polyodon spathula*. *J. Appl. Ichthyol.* **28**, 341–345 (2012). 26566297 10.1111/j.1439-0426.2012.01976.x PMC4640176

[24] Gillis, J. A., Dahn, R. D. & Shubin, N. H. Chondrogenesis and homology of the visceral skeleton in the little skate, *Leucoraja erinacea* (Chondrichthyes: Batoidea). *J. Morphol.* **270**, 628–643. 10.1002/jmor.10710 (2009). 19117064 10.1002/jmor.10710

[25] Warth, P., Hilton, E. J., Naumann, B., Olsson, L. & Konstantinidis, P. Development of the skull and pectoral girdle in Siberian sturgeon, *Acipenser baerii*, and Russian sturgeon, *Acipenser gueldenstaedtii* (A cipenseriformes: A cipenseridae). *J. Morphol.* **278**, 418–442 (2017). 28176372 10.1002/jmor.20653

[26] Langille, R. M. & Hall, B. K. Development of the head skeleton of the Japanese medaka, *Oryzias latipes* (Teleostei). *J. Morphol.* **193**, 135–158 (1987). 29907002 10.1002/jmor.1051930203

[27] Dutel, H. *et al.* Neurocranial development of the coelacanth and the evolution of the sarcopterygian head. *Nature* **569**, 556–559. 10.1038/s41586-019-1117-3 (2019). 30996349 10.1038/s41586-019-1117-3

[28] Kemp, A. Ontogeny of the skull of the Australian lungfish *Neoceratodus forsteri* (Osteichthyes: Dipnoi). *J. Zool.* **248**, 97–137 (1999). 10.1111/j.1469-7998.1999.tb01027.x

[29] Gaupp, E. *Die Entwicklung des Kopfskelettes, Kap 6* Vol. Bd 3 (Verlag von Gustav Fischer, 1905).

[30] De Beer, G. *The development of the vertebrate skull* (Oxford University Press, 1937).

[31] Hernández-Jaimes, C., Jerez, A. & Ramírez-Pinilla, M. P. Embryonic development of the skull of the Andean lizard *Ptychoglossus bicolor* (Squamata, Gymnophthalmidae). *J Anat* **221**, 285–302 (2012). 22881276 10.1111/j.1469-7580.2012.01549.x PMC3458248

[32] Ollonen, J., Da Silva, F. O., Mahlow, K. & Di-Poï, N. Skull development, ossification pattern, and adult shape in the emerging lizard model organism *Pogona vitticeps*: A comparative analysis with other squamates. *Front. Physiol.* **9**, 278 (2018). 29643813 10.3389/fphys.2018.00278 PMC5882870

[33] Yaryhin, O. & Werneburg, I. Tracing the developmental origin of a lizard skull: Chondrocranial architecture, heterochrony, and variation in lacertids. *J. Morphol.* **279**, 1058–1087 (2018). 29882601 10.1002/jmor.20832

[34] Kuratani, S. Development of the chondrocranium of the loggerhead turtle, *Caretta caretta*. *Zool. Sci.* **16**, 803–818 (1999). 10.2108/zsj.16.803

[35] Tulenko, F. J. & Sheil, C. A. Formation of the chondrocranium of *Trachemys scripta* (Reptilia: Testudines: Emydidae) and a comparison with other described turtle taxa. *J. Morphol.* **268**, 127–151 (2007). 17236189 10.1002/jmor.10487

[36] Müller, F. Zur embryonalen Kopfentwicklung von" Crocodylus cataphractus" Cuv. *Rev Suisse Zool.* **74**, 189–294. 10.5962/bhl.part.75851 (1967). 5615286 10.5962/bhl.part.75851

[37] Vieira, L. G. *et al.* Ontogeny of the skull of the Black Caiman (*Melanosuchus niger*) (Crocodylia: Alligatoridae). *Can. J. Zool.* **97**, 142–155 (2019). 10.1139/cjz-2018-0076

[38] Hüppi, E., Werneburg, I. & Sánchez-Villagra, M. R. Evolution and development of the bird chondrocranium. *Front. Zool.* **18**, 1–27 (2021). 33926502 10.1186/s12983-021-00406-z PMC8082637

[39] Lukas, P., Araújo, O. G. S. & Hernandez-Nieto, S. The chondrocranium of the tadpole of *Hymenochirus boettgeri* and the sequence and timing of its development. *Zoologischer Anzeiger* **310**, 53–66 (2024). 10.1016/j.jcz.2024.04.002

[40] Parker, W. K. XXIV. On the structure and development of the skull in the batrachia—Part II. *Philosophical Transactions of the Royal Society of London*, 601–669 (1876).

[41] Parker, W. On the structure and development of the skull in the batrachian. Part III. *Proc. R. Soc. Lond.* **30**, 435–438 (1879).

[42] Stöhr, P. Zur Entwickelungsgeschichte des Anurenschädels. *Zeitschrift für Wissenschaftliche Zoologie* **36**, 68–103 (1876).

[43] Stephenson, N. On the development of the chondrocranium and visceral arches of *Leiopelma archeyi*. *Trans. Zool. Soc. Lond.* **27**, 203–253 (1951). 10.1111/j.1096-3642.1951.tb00229.x

[44] Van der Westhuizen, C. M. The development of the chondrocranium of *Heleophryne purcelli* Sclater with special reference to the palatoquadrate and the sound-conducting apparatus. *Acta Zool.* **42**, 1–72 (1961). 10.1111/j.1463-6395.1961.tb00059.x

[45] Frost, D. R. *et al.* The amphibian tree of life. *Bull. Am. Mus. Nat. Hist.* **2006**, 1–291 (2006). 10.1206/0003-0090(2006)297[0001:TATOL]2.0.CO;2

[46] Ziermann, J. M., Freitas, R. & Diogo, R. Muscle development in the shark *Scyliorhinus canicula*: Implications for the evolution of the gnathostome head and paired appendage musculature. *Front. Zool.* **14**, 31. 10.1186/s12983-017-0216-y (2017). 28649268 10.1186/s12983-017-0216-y PMC5480186

[47] Ziermann, J. M., Mitgutsch, C. & Olsson, L. Analyzing developmental sequences with Parsimov-a case study of cranial muscle development in Anuran Larvae. *J. Exp. Zool. Part B Mol. Dev. Evol.* **322**, 586–606. 10.1002/jez.b.22566 (2014). 10.1002/jez.b.22566 24692269

[48] Duellman, W. E. & Trueb, L. *Biology of Amphibians* (Johns Hopkins University Press, 1994).

[49] Klein, P. Early development of *Xenopus laevis*: A laboratory manual. *Q Rev Biol.* **76**, 1–236 (2001). 10.1086/393914

[50] Gosner, K. L. A simplified table for staging anuran embryos and larvae with notes on identification. *Herpetologica (Herpetologists’ League, Allen Press, Lawrence)* **16**, 183–190 (1960).

[51] Heidenhain, M. Ueber die Mallorysche Bindegewebesfaerbung mit Karmin und Azokarmin als Vorfarben. *Z Wiss Mikrosk Mikrosk Tech* **32**, 361–372 (1915).

[52] Dingerkus, G. & Uhler, L. D. Enzyme clearing of alcian blue stained whole small vertebrates for demonstration of cartilage. *Stain Technol.* **52**, 229–232. 10.3109/10520297709116780 (1977). 71769 10.3109/10520297709116780

[53] Schindelin, J. *et al.* Fiji: an open-source platform for biological-image analysis. *Nature methods* **9**, 676–682 (2012). 22743772 10.1038/nmeth.2019 PMC3855844

[54] Cardona, A. *et al.* TrakEM2 software for neural circuit reconstruction. *PLoS One* **7**, e38011 (2012). 22723842 10.1371/journal.pone.0038011 PMC3378562

[55] Haas, A. Phylogeny of frogs as inferred from primarily larval characters (Amphibia: Anura). *Cladistics* **19**, 23–89. 10.1016/s0748-3007(03)00006-9 (2003). 34905866 10.1016/s0748-3007(03)00006-9

[56] Trueb, L. & Hanken, J. Skeletal development in *Xenopus laevis* (Anura: Pipidae). *J. Morphol.* **214**, 1–41 (1992). 1433306 10.1002/jmor.1052140102

